# Supplementary material for: Bio-Based Packaging Materials Containing Substances Derived from Coffee and Tea Plants
Source: Materials (Basel). 2020 Dec 15;13(24):5719. doi: 10.3390/ma13245719 (PMC7765424; doi:10.3390/ma13245719)
Supplement: Supplementary file 1 [file materials-13-05719-s001.pdf]

materials

Supplementary Material

Bio-Based Packaging Materials Containing Substances Derived from Coffee and Tea Plants

Olga Olejnik and Anna Masek *

Institute of Polymer and Dye Technology, Lodz University of Technology, ul. Stefanowskiego 12/16, 90-924 Lodz, Poland; olejnik.olg@gmail.com

* Correspondence: anna.masek@p.lodz.pl

Received: 3 November 2020; Accepted: 8 December 2020; Published: date

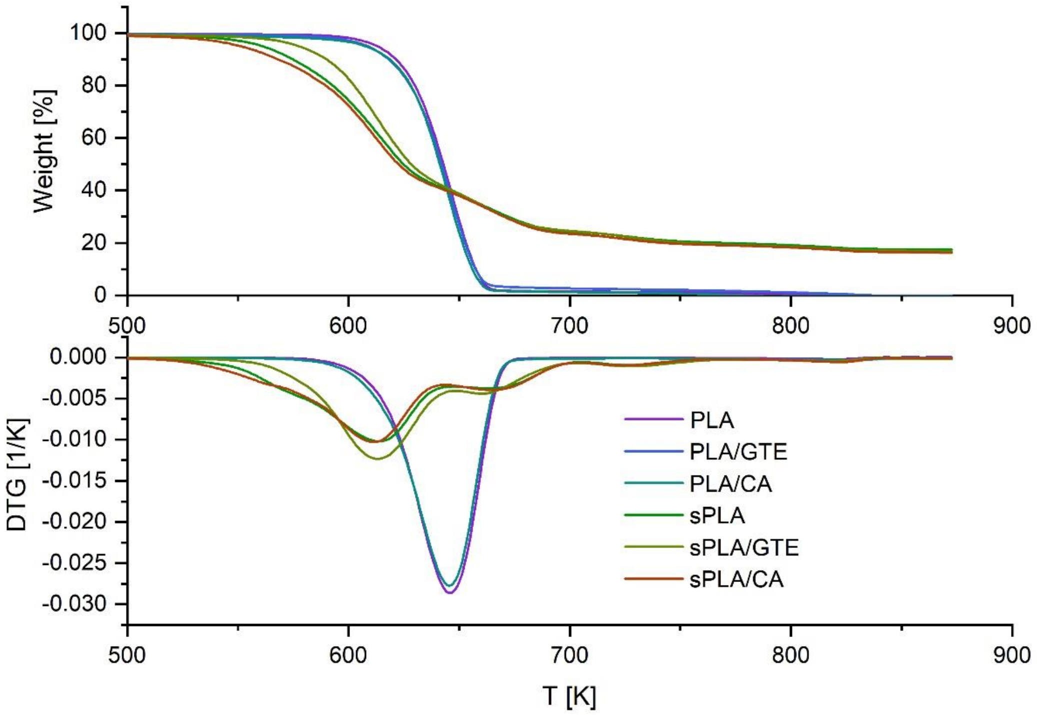

Figure S1. Thermogravimetric curves of PLA and Bioplast with polyphenon 60 and caffeic acid.

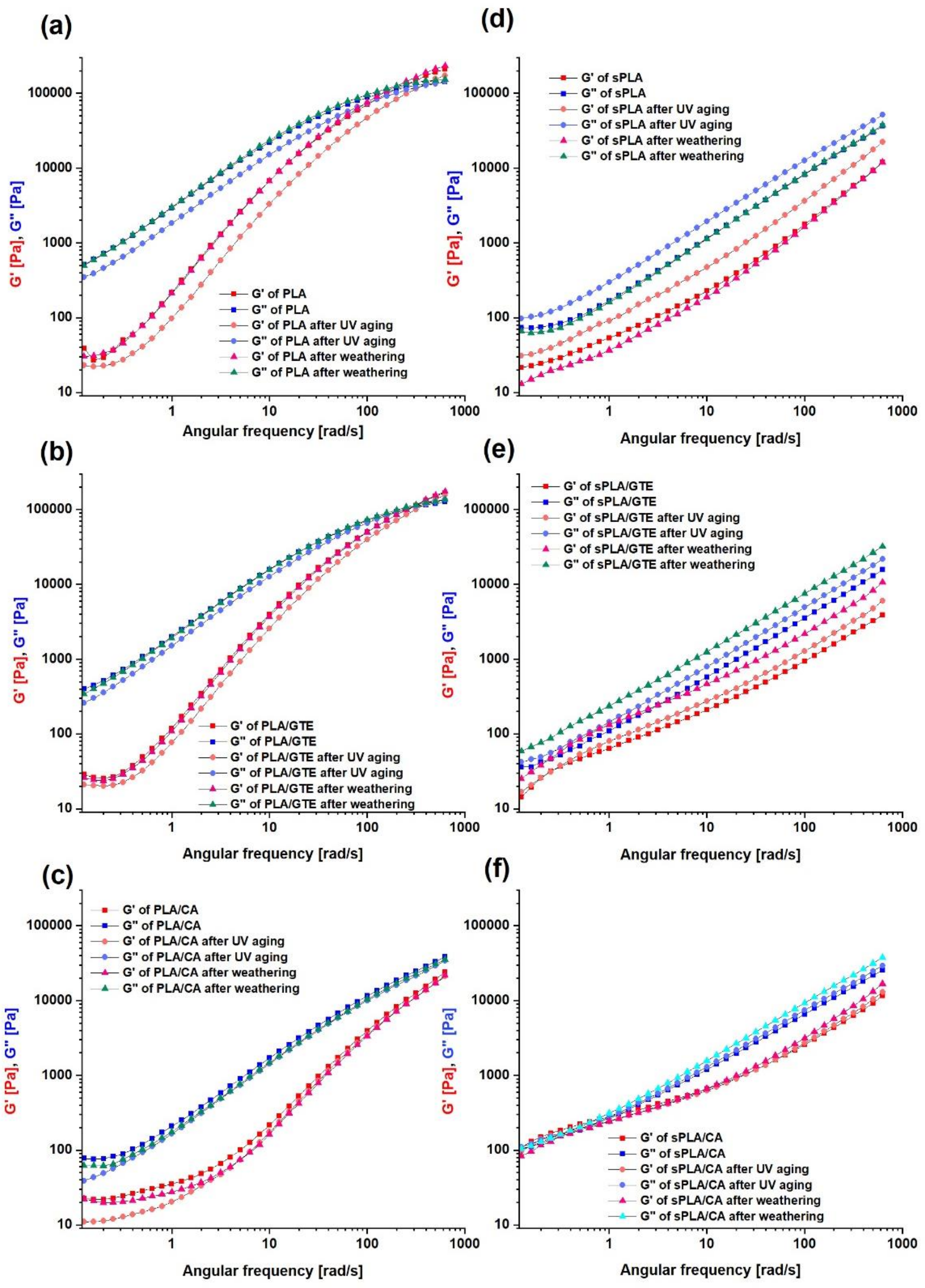

Figure S2. Storage modulus (G’), loss modulus (G’’) of: (**a**) polylactide (PLA), (**b**) polylactide with green tea extract (PLA/GTE), (**c**) polylactide with caffeic acid (PLA/CA), (**d**) polylacide containing starch (sPLA), (**e**) polylactide containing starch with green tea extract (sPLA/GTE), (**f**) polylactide containing starch with caffeic acid (sPLA/CA) in a function of angular frequency before and after different aging types.

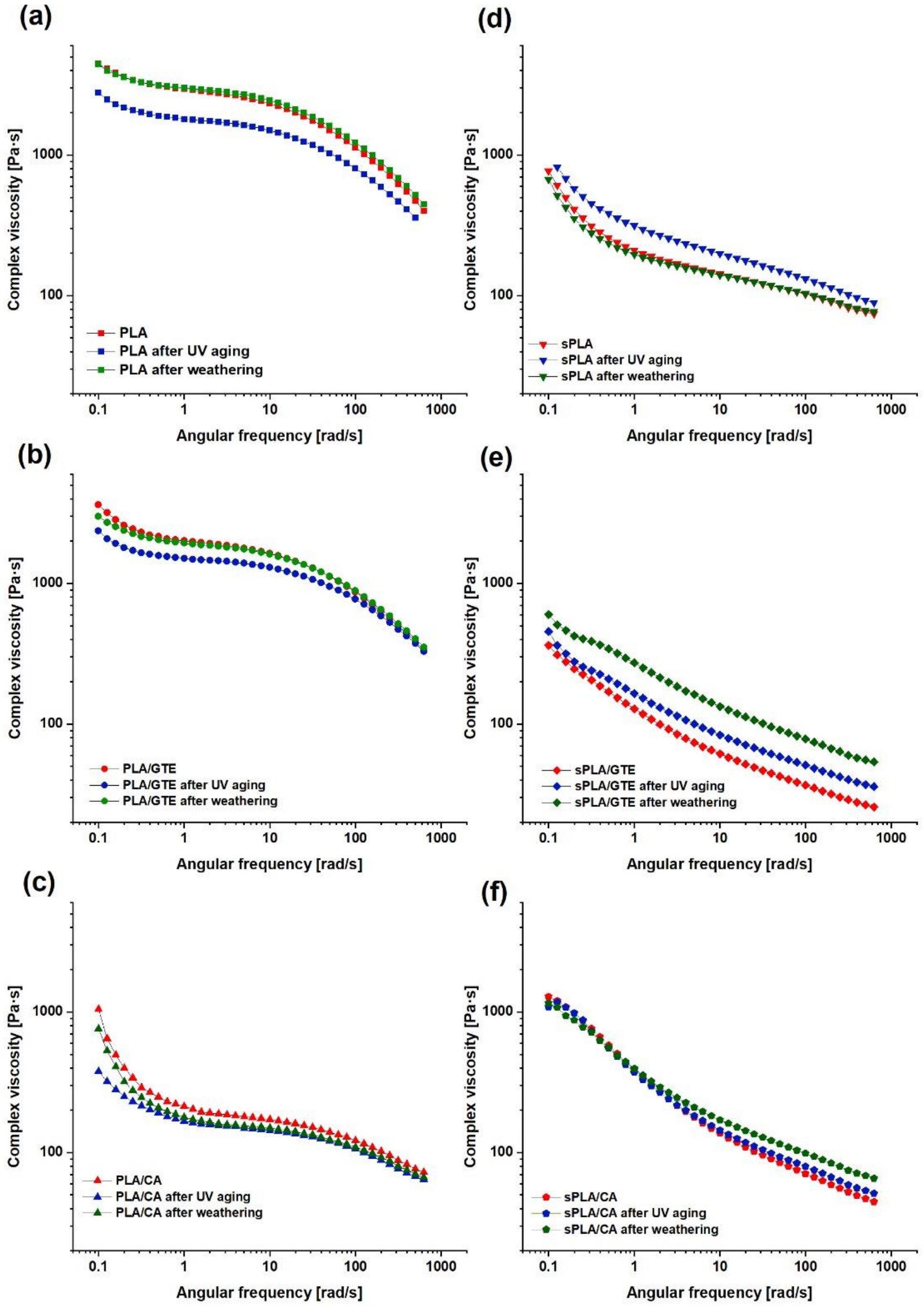

Figure S3. Complex viscosity [Pa·s] of: (**a**) polylactide (PLA), (**b**) polylactide with green tea extract (PLA/GTE), (**c**) polylactide with caffeic acid (PLA/CA), (**d**) polylacide containing starch (sPLA), (**e**) polylactide containing starch with green tea extract (sPLA/GTE), (**f**) polylactide containing starch with caffeic acid (sPLA/CA) in a function of angular frequency before and after different aging types.

© 2020 by the authors. Licensee MDPI, Basel, Switzerland. This article is an open access article distributed under the terms and conditions of the Creative Commons Attribution (CC BY) license (http://creativecommons.org/licenses/by/4.0/).
